# Editorial: Unlocking autophagy’s full potential: embracing a multidimensional approach for targeted cancer treatment

**DOI:** 10.3389/fphar.2026.1812809

**Published:** 2026-03-09

**Authors:** Valerie R. Wiersma

**Affiliations:** Department of Hematology, University Medical Center Groningen, University of Groningen, Groningen, Netherlands

**Keywords:** autophagy, cancer, cell death, chemotherapy, immunotherapy, therapeutic targeting

**FIGURE 1:**
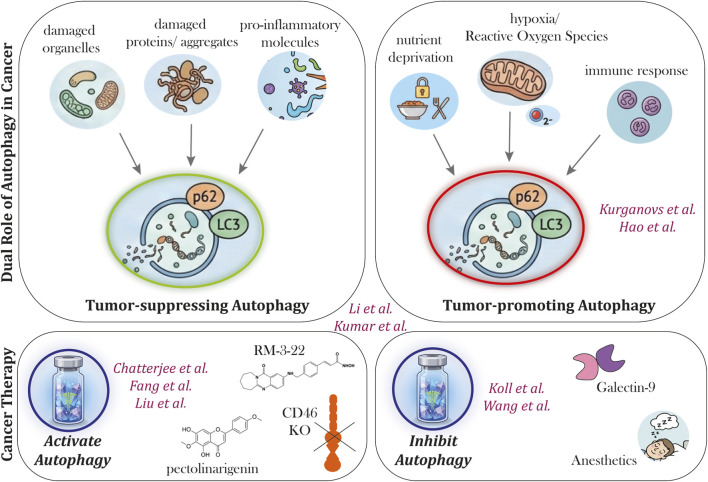
Unlocking Autophagy’s full potential: embracing a multidimensional approach for targeted cancer treatment. Autophagy has a dual role in cancer, known to be tumor-suppressing at the pre-cancerous stage and tumor-promoting in established tumors. Hence, targeting of the autophagy pathway for cancer therapy can comprise both the activation and the inhibition of autophagy. All papers included in this research topic are shown in purple, and specifically placed in the figure based on their content.

Autophagy, an evolutionarily conserved cellular process, plays a pivotal role in maintaining cellular homeostasis by facilitating the degradation of damaged or dysfunctional cellular components. Perturbations in autophagy have been associated with various pathological conditions, including cancer (Folkerts et al., 2019). Therefore, targeting autophagy is of interest for the treatment of cancer, which was the focus of this research topic (Figure 1).

The importance of autophagy in cancer was highlighted in the review of Kurganovs et al. that focused on the role of autophagy during the development of prostate cancer, and described how autophagy impacts on the therapeutic response. Autophagy is also of importance in lung cancer as described by Hao et al. who developed a mouse model (*Rb1* ^*fl/fl*^; *Trp53* ^*fl/fl*^; GFP-LC3-RFP-LC3△G) that enabled *in vivo* tracking of autophagy. Using this model, it was demonstrated that lung tumor subpopulations with high autophagic flux displayed increased proliferation and enhanced metastatic potential.

Indeed, autophagy is widely considered as a pro-survival mechanism, especially for cells under stress, among which cancer cells. This argues for the inhibition of autophagy as a promising strategy for cancer therapy. In this respect, Koll et al. demonstrated that the glycan-binding protein Galectin-9 was cytotoxic for malignant B cells by inhibiting the proper execution of autophagy. Here, malignant B cell lines with lower LC3B-I expression levels were more sensitive towards Galectin-9 treatment. This data suggests that Galectin-9 sensitivity is related to basal levels of autophagy flux, whereby cells that rely more on autophagy for their survival are more sensitive to the inhibition of this pathway. However, autophagy can also be a cell death pathway itself. In this respect, Chatterjee et al. demonstrated that the activation of the autophagy pathway preceded the activation of apoptosis and subsequent cell death when lung cancer cells were treated with RM-3-22, a novel TAZQ-based hydroxamic acid derivative with histone deacetylase inhibitory activity. Correspondingly, 3-MA, an autophagy inhibitor, prevented cleavage of PARP-1, which is an event in apoptosis activation. Also *in vivo*, treatment with RM-3-22 reduced tumour growth, demonstrating potency of autophagy activation for lung cancer therapy. Similar results were reported by Fang et al. who demonstrated that pectolinarigenin had anti-cancer effects towards cervical cancer both *in vitro* and *in vivo*. Treatment with pectolinarigenin activated the apoptosis pathway as evidenced by the activation of Bax and subsequent caspase-3 cleavage. Also LC3B-II was induced, indicative of activated autophagy. Indeed, 3-MA inhibited the effects of pectolinarigenin. Thus either the inhibition or activation of autophagy can be exploited for cancer therapy. In this respect, the role of autophagy may be opposing at various stages of tumorigenesis and during cancer progression, requiring different strategies for therapeutic modulation of autophagy, as was also reviewed by Kumar et al..

Autophagy is regulated at multiple levels by various pathways. Liu et al. described the relation between CD46 and autophagy in oral squamous cell carcinoma, whereby elevated expression of CD46 (compared to healthy cells) associated with low levels of the autophagy markers LC3B and ATG5. Correspondingly, the KO of CD46 increased the expression of autophagy-related as well as apoptosis-related genes. These effects of CD46-KO were seen both *in vitro* and *in vivo*, and were associated with cytotoxicity and reduced tumor volume. Also less apparent factors can impact on autophagy. In this respect, Wang et al. described the effect of anesthetics on autophagy, which may impact on tumor progression. Furthermore, autophagy is modulated by non-coding-RNAs as was review by Li et al. in the context of lung cancer. Hence, autophagy may also be indirectly modulated for cancer therapy by targeting these non-coding-RNAs or molecules like CD46.

Together, autophagy is a multifaceted pathway and promising target for cancer therapy. Although not part of this research topic, autophagy-modulation may also be used to steer anti-cancer immune responses, as also highlighted in a recent research topic of Frontiers (Gustavo et al., 2025). Interestingly, both the modulation at the cancer side, as well as the regulation at the immune effector cell side can improve anti-cancer immune responses. Recently, this has also been demonstrated for chimeric antigen receptor (CAR) T cell therapy, a relatively novel immunotherapeutic strategy that revolutionized the landscape of cancer immunotherapy. Specifically, activation of autophagy in the CAR T cells improved their persistence and anti-tumor activity (Akhtar et al., 2025), whereas inhibition of autophagy in the cancer cells made them more vulnerable to CAR T cell-induced cytotoxicity (De Mitri et al., 2025; Tang et al., 2024).

However, an issue that still needs to be addressed to enable full clinical translation is the current lack of autophagy modulators and especially inhibitors that are suitable for clinical use. Chloroquine and its derivative hydroxychloroquine are clinically approved autophagy drugs with reasonable safety profiles, but their clinical efficacy in cancer therapy remains limited (Jain et al., 2023). Numerous novel autophagy inhibitors have been developed over the past decade, but their systemic use is commonly hampered by unwanted side-effects. Hence, strategies for cancer-specific targeted delivery of autophagy inhibitors are of interest to be further developed (Mahri et al., 2025). In addition, characterizing autophagy-modulating capacities of already clinically approved drugs may be useful to accelerate the implementation of autophagy targeting into clinical practice (Lin et al., 2023).

Thus, as highlighted by this research topic, autophagy is a versatile pathway that is a promising target for cancer therapy (Figure 1). Future research should however be focused on the development of more specific modulators and/or modalities for targeted delivery, to advance autophagy modulation as a therapeutic strategy in cancer therapy.
